# A better understanding of the association between maternal perception of foetal movements and late stillbirth—findings from an individual participant data meta-analysis

**DOI:** 10.1186/s12916-021-02140-z

**Published:** 2021-11-15

**Authors:** John M. D. Thompson, Jessica Wilson, Billie F. Bradford, Minglan Li, Robin S. Cronin, Adrienne Gordon, Camille H. Raynes-Greenow, Tomasina Stacey, Vicki M. Cullling, Lisa M. Askie, Louise M. O’Brien, Edwin A. Mitchell, Lesley M. E. McCowan, Alexander E. P. Heazell

**Affiliations:** 1 Department of Obstetrics and Gynaecology, Faculty of Medical and Health Sciences, University of Auckland, Private Bag 92019, Auckland, 1042 New Zealand; 2 Department of Paediatrics: Child and Youth Health, Faculty of Medical and Health Sciences, University of Auckland, Auckland, New Zealand; 3 School of Nursing, Midwifery and Health Practice, Victoria University of Wellington, Wellington, New Zealand; 4 Discipline of Obstetrics, Gynaecology and Neonatology, University of Sydney, Sydney, Australia; 5 Sydney School of Public Health, University of Sydney, Sydney, Australia; 6 Department of Nursing and Midwifery, School of Human and Health Sciences, University of Huddersfield, Huddersfield, England UK; 7 National Health and Medical Research Council Clinical Trials Centre, University of Sydney, Sydney, Australia; 8 Departments of Neurology Sleep Disorders Center, University of Michigan, Ann Arbor, MI USA; 9 Obstetrics and Gynecology, University of Michigan, Ann Arbor, MI USA; 10 Division of Developmental Biology & Medicine, Maternal and Fetal Health Research Centre, School of Medical Sciences, University of Manchester, Manchester, England UK

**Keywords:** Stillbirth, Foetal death, Foetal movements, Decreased foetal movements, Individual participant data meta-analysis, Hiccups, Vigorous movement

## Abstract

**Background:**

Late stillbirth continues to affect 3–4/1000 pregnancies in high-resource settings, with even higher rates in low-resource settings. Reduced foetal movements are frequently reported by women prior to foetal death, but there remains a poor understanding of the reasons and how to deal with this symptom clinically, particularly during the preterm phase of gestation. We aimed to determine which women are at the greatest odds of stillbirth in relation to the maternal report of foetal movements in late pregnancy (≥ 28 weeks’ gestation).

**Methods:**

This is an individual participant data meta-analysis of all identified case-control studies of late stillbirth. Studies included in the IPD were two from New Zealand, one from Australia, one from the UK and an internet-based study based out of the USA. There were a total of 851 late stillbirths, and 2257 controls with ongoing pregnancies.

**Results:**

Increasing strength of foetal movements was the most commonly reported (> 60%) pattern by women in late pregnancy, which were associated with a decreased odds of late stillbirth (adjusted odds ratio (aOR) = 0.20, 95% CI 0.15 to 0.27). Compared to no change in strength or frequency women reporting decreased frequency of movements in the last 2 weeks had increased odds of late stillbirth (aOR = 2.33, 95% CI 1.73 to 3.14). Interaction analysis showed increased strength of movements had a greater protective effect and decreased frequency of movements greater odds of late stillbirth at preterm gestations (28–36 weeks’ gestation). Foetal hiccups (aOR = 0.45, 95% CI 0.36 to 0.58) and regular episodes of vigorous movement (aOR = 0.67, 95% CI 0.52 to 0.87) were associated with decreased odds of late stillbirth. A single episode of unusually vigorous movement was associated with increased odds (aOR = 2.86, 95% CI 2.01 to 4.07), which was higher in women at term.

**Conclusions:**

Reduced foetal movements are associated with late stillbirth, with the association strongest at preterm gestations. Foetal hiccups and multiple episodes of vigorous movements are reassuring at all gestations after 28 weeks’ gestation, whereas a single episode of vigorous movement is associated with stillbirth at term.

**Supplementary Information:**

The online version contains supplementary material available at 10.1186/s12916-021-02140-z.

## Background

The sensation of foetal movements from mid-pregnancy onwards is interpreted as a sign of foetal well-being [1]. Conversely, changes in foetal movements, particularly when they reduce or become absent, are a cause of maternal concern and have been associated with an increased risk of poor outcomes including preterm birth, small for gestational age infants, late stillbirth and neurodevelopmental delay [2]. However, translating this information into practical advice for women is complex, because each pregnancy is different, there are no robustly determined ‘alarm limits’ [3], and intervention has the potential for inadvertent harm [4].

A number of case-control studies have examined the changes in frequency and strength of maternal perception of foetal movements in the third trimester [5–7]. The Auckland Stillbirth Study (TASS) [7] found no increased risk of late stillbirth associated with a decrease in frequency or decrease in strength of foetal movements in women after 37 weeks’ gestation. Conversely, increasing strength or frequency of movements in late pregnancy was associated with a reduction in late stillbirth. A confirmatory multicentre case-control study in New Zealand [5] and a larger study in the UK identified similar prevalence’s and effect sizes [6].

Two recent cluster randomised control trials (cRCT) have been conducted, the ‘AFFIRM’ trial [4] and ‘Mindfetalness’ [8], found no difference in their primary outcomes of perinatal death and proportion of Apgar scores < 7, respectively. A systematic review and meta-analysis of RCTs which included 468,601 pregnancies (82% from the AFFIRM study) reported a small reduction in perinatal deaths [9]. However, this systematic review was limited to univariable analyses, and the AFFIRM trial which dominates this review showed the risk moved towards unity after adjustment for potential confounders [4]. The lack of conclusive findings from these large RCTs has led to uncertainty about how to address and manage reduced foetal movements in clinical practice.

Furthermore, the relationship between other aspects of foetal activity and late stillbirth is unclear. The case-control studies identified the common presence of hiccups, with over 60% of all control women reporting feeling hiccups in the last 2 weeks and reported decreased odds of late stillbirth with this sensation. However, in an internet-based case-control study using a self-completed online questionnaire, frequent hiccups were reported by high proportions of both women who had a late stillbirth and women whose infants were live born [10]. This study also reported that a single episode of vigorous movements followed by cessation of movements was associated with late stillbirth, which the authors attributed to the timing of the demise of the infant [11].

Whilst the association between increased strength of movements over a discrete time period in late pregnancy and the reduced risk of late stillbirth is consistent across studies, the effect of other aspects of foetal movements over the same period of time is less clear. In addition, it is uncertain whether such associations are consistent in different groups of women. For example, women with obesity are more likely to present to their care provider with RFM [12]; their concerns may be dismissed because of an assumption that perception of RFM movements is due to their body size [13]. A systematic review of the literature supported these suggestions but found that there was limited evidence of the association of increased maternal BMI, RFM and outcomes [14]. However, available data from a study that included 233 obese women suggested that women with obesity feel changes in strength and frequency in foetal movements in the same proportion as non-obese women [15]. To optimise the quality of information available from the case-control studies and evaluate the influence of confounding factors, we established the Collaborative Individual Participant Data (IPD) Meta-analysis of Sleep and Stillbirth (CRIBSS). It was anticipated that a better understanding of the association of altered foetal activity and late stillbirth would contribute to the interpretation of clinical trials in this field.

This study was a planned secondary analysis of the CRIBSS data set [16]; the specific aims of this analysis were as follows:

1. To determine the association of the frequency and strength of foetal movements, the presence of hiccups, uterine contractions and the frequency of vigorous movements in the third trimester in relation to late stillbirth
2. To determine whether associations of foetal movements and hiccups differ in terms of gestational age at interview
3. To determine whether associations of foetal movements and hiccups differ in terms of whether women are obese

## Methods

### Patient involvement

Representatives of parent support groups from Australia (Stillbirth Foundation Australia) and New Zealand (SANDS) were involved in the development and conceptualisation of the IPD protocol and analyses.

This IPD includes several case-control studies with cases of late stillbirth and women with ongoing pregnancies (controls) that have been harmonised into the CRIBSS data set. The study was developed and registered according to the guidelines of the PROSPERO register of systematic reviews (CRD42107047703), and the protocol paper which describes in detail the processes for searches and study eligibility [16] and further details of the studies included have been published in previous analyses, including the extensive description of the eligible studies and risk of bias assessment [17, 18], and in brief in Additional file 1: Table S1. The CRIBBS data set includes data from five case-control studies: two from New Zealand (The Auckland Stillbirth Study (TASS) and the Multi-Centre Stillbirth Study (MCSS)) [7, 19], one from Australia (Sydney Stillbirth Study (SSS)) [20], one from the UK (The Midlands and North East Stillbirth Study (MINESS)) [21], and an international survey (STARS) [22].

Cases were women who delivered a singleton, non-anomalous stillborn infant at or after 28 weeks’ gestation. In three studies, controls were women with an ongoing pregnancy, gestation-matched to the expected distribution of gestational age of stillbirths in participating maternity units over the previous 3–4 years [7, 19, 21]. Controls for one study were matched for booking hospital and expected delivery date [20], and the internet-based study simply included women with ongoing pregnancies over 28 weeks’ gestation [22]. Interviewer-administered questionnaires were used in all studies other than the internet-based study, which was completed online. Information was collected in all studies within a median of 6 weeks of stillbirth or at a similar gestation for controls. Harmonisation of data across the studies was carried out by aligning responses to similar questions that were used across studies.

Data on changes in foetal movements were assessed through questions about strength and frequency of movements and whether these had increased, decreased or remained the same over the last 2 weeks. An option of unsure was also available. A prioritised strength-frequency variable was created based on the prevalence of responses in each variable and previous publications [5, 6]. The order of priority was increased strength, increased frequency, decreased frequency, no change and unsure. Vigorous foetal movement variables were defined as more than usual vigorous movements in the last 2 weeks (yes, no, unsure), and if yes, whether this was a single episode or more than once. Data on hiccups was assessed by asking whether women had felt their baby having hiccups in the last 2 weeks with the options of yes, no and unsure. Information was also collected on whether women had felt uterine contractions or not in the last 2 weeks (yes/no).

For the interaction analyses, gestational age at interview was categorised into three groups, very preterm (28–32 weeks), preterm (33–36 weeks) and term (37+ weeks). Pre-pregnancy or earliest recorded weight and self-reported height were used to calculate body mass index (BMI), then categorised as obese (BMI ≥ 30) or non-obese (BMI < 30).

### Statistical analysis

The variables of primary interest in the analysis were (1) patterns (strength and frequency) of foetal movements as described by the mother over the last 2 weeks and the prioritised strength-frequency variable, (2) episodes of vigorous movements, (3) the feeling of foetal hiccups and (4) the feeling of uterine contractions.

The IPD analysis was carried out using a one-stage approach, such that data from each of the participating studies were included in a single model. Logistic regression models were used for the binary outcome (late stillbirth). A fixed study effect and a study site effect were included in the model specification as strata (see Additional file 2 for example). The process of analysis progressed in a stepwise manner; firstly, univariable analyses were performed. Secondly, each foetal-movement variable was added to a pre-defined multivariable model that controlled for maternal age, earliest pregnancy BMI, maternal ethnicity, parity, maternal education level, marital status, pre-existing hypertension or diabetes, maternal smoking, recreational drug use, supine going to sleep position and customised birthweight centile for each country (https://www.gestation.net/). Finally, a multivariable model controlling for the above potential confounders and all four foetal movement variables was fitted. Additional sensitivity analysis was carried out excluding the STARS, which had missing control data on foetal movements and customised centile data.

All analyses were performed using unconditional logistic regression using the logistic procedure in SAS v9.4 (SAS Institute, Cary, NC), to estimate the odds for stillbirth associated with maternally reported perception of foetal movements. Statistical significance was defined at the 5% level.

## Results

This analysis included 851 cases of late stillbirth and 2257 control women of similar gestational age. Women were from across the reproductive age range, and the majority were Caucasian (Table 1). A descriptive analysis of foetal movement variables included in the one-stage analysis is presented in Table 1, and a breakdown of the same variables by individual study is available in Additional file 1: Table S2.

**Table 1:** Demographic characteristics of cases and controls in the IPD analysis

Variable	***N*** = 851	Cases, ***n*** or mean	% or ***s.d.***	***N*** = 2257	Controls, ***n*** or mean	% or ***s.d.***	Chi-square or ***t***-test (***p***-value)
**Maternal age**							12.24 (0.06)
< 20	846	33	3.9	2238	59	2.6	
20–24		115	13.6		274	12.2	
25–29		228	27.0		631	28.2	
30–34		265	31.3		794	35.5	
35–39		160	18.9		396	17.7	
40+		45	5.3		84	3.8	
**Maternal BMI**	842	27.7	6.9	2229	26.3	6.1	4.99 (< 0.0001)
**Maternal ethnicity**							21.43 (0.0015)
White	851	522	61.3	2257	1545	68.5	
Black		22	2.6		42	1.9	
South Asian		90	10.6		219	9.7	
South East and East Asian		40	4.7		111	4.9	
Maori		46	5.4		107	4.7	
Pacific		91	10.7		154	6.8	
Others		40	4.7		79	3.5	
**Parity**							56.49 (< 0.0001)
Nulliparous	851	446	52.4	2257	930	41.2	
1–2		292	34.3		1110	49.2	
3–4		87	10.2		176	7.8	
5+		26	3.1		41	1.8	
**Maternal education**							39.59 (< 0.0001)
Primary	842	187	22.2	2249	348	15.5	
Secondary		161	19.1		343	15.3	
University		328	39.0		1069	47.5	
Post-graduate degree		73	8.7		240	10.7	
Non-university trading education		93	11.0		249	11.1	
**Marital status**							22.60 (< 0.0001)
Not in stable relationship	847	92	10.9	2255	143	6.3	
Stable relationship		755	89.1		2112	93.7	
**Smoking status**							39.19 (< 0.0001)
Current smoker	848	145	17.1	2247	205	9.1	
Non-smoker or quit smoking before end of 1st trimester		703	82.9		2042	90.9	
**Gestational age (weeks)**	851	36.7	3.6	2257	36.3	3.7	2.64 (0.008)
28–32		151	17.7		479	21.2	5.78 (0.06)
33–36		214	25.2		523	23.2	
37+		486	57.1		1255	55.6	

### Association of foetal movements and stillbirth

We found a 78% decrease in late stillbirth associated with the increased strength of movements in the univariable analysis (OR = 0.22 (95% CI 0.17–0.27)), which remained consistent in the multivariable analysis (adjusted odds ratio (aOR) = 0.20, 95% CI 0.15–0.27). The association of an increase in frequency where strength was not increased also showed decreased odds (aOR = 0.50 (95% CI 0.29–0.88)), but not of the same magnitude as that of strength. Conversely, a decrease in the frequency of movements was associated with an increased odds of late stillbirth (aOR = 2.33, 95% CI 1.73–3.14) (Table 2).

**Table 2:** Univariable and multivariable odds ratios showing the association between foetal movement variables and late stillbirth

	Case, ***N*** = 851	Control, ***N*** = 2257	Univariable OR (95% CI)	Adjusted OR* (95% CI)	Multivariable OR** (95% CI)
**Strength of movements in the last 2 weeks: 4 categories**					
Increased	124	1061	**0.21 (0.17, 0.26)**	**0.18 (0.14, 0.23)**	
Decreased	195	179	**1.94 (1.53, 2.45)**	**1.82 (1.37, 2.42)**	
No change	446	793	1	1	
Unsure	79	118	1.19 (0.88, 1.62)	0.96 (0.67, 1.38)	
**Frequency of movements in the last 2 weeks: 4 categories**					
Increased	83	648	**0.33 (0.25, 0.42)**	**0.29 (0.21, 0.38)**	
Decreased	265	268	**2.52 (2.06, 3.09)**	**2.48 (1.94, 3.16)**	
No change	443	1131	1	1	
Unsure	53	114	1.19 (0.84, 1.67)	0.93 (0.62, 1.39)	
**How often was a baby more vigorous than usual in the last 2 weeks: 3 categories**					
Once	133	124	**2.25 (1.72, 2.95)**	**2.30 (1.68, 3.16)**	**2.86 (2.01, 4.07)**
More than once	165	903	**0.38 (0.31, 0.47)**	**0.41 (0.32, 0.52)**	**0.67 (0.52, 0.87)**
Never	430	901	1	1	1
**During the last 2 weeks/this pregnancy did you feel your baby having hiccups?**					
Yes	355	1359	**0.40 (0.34, 0.48)**	**0.42 (0.34, 0.53)**	**0.45 (0.36, 0.58)**
No	329	509	1	1	1
Unsure	51	100	0.79 (0.55, 1.14)	0.94 (0.61, 1.44)	0.93 (0.58, 1.49)
**During the last 2 weeks, did you feel uterine contractions (tightenings/pre-labour contractions/Braxton-Hicks contractions/false labour)?**					
Yes	253	653	1.04 (0.86, 1.25)	0.97 (0.77, 1.22)	
No	352	941	1	1	
**Combination of strength and frequency changes in the last 2 weeks (prioritised variable)**	Missing = 6	Missing = 95			
Increased strength	124	1061	**0.22 (0.17, 0.27)**	**0.19 (0.15, 0.24)**	**0.20 (0.15, 0.27)**
Increased frequency but not strength	25	97	**0.47 (0.30, 0.75)**	**0.44 (0.26, 0.74)**	**0.50 (0.29, 0.88)**
Decreased frequency	249	196	**2.34 (1.87, 2.92)**	**2.36 (1.79, 3.10)**	**2.33 (1.73, 3.14)**
Unsure of strength or frequency	57	91	1.15 (0.81, 1.64)	1.01 (0.67, 1.52)	0.85 (0.53, 1.38)
The same strength or frequency	390	717	1	1	1

Adjusted and multivariable models adjusted for maternal age (6 groups), maternal BMI (continuous), maternal ethnicity (7 groups), parity (4 groups), maternal education (5 groups), marital status (yes/no), maternal pre-existing HTN, DM (yes/no), smoking status (yes/no), recreation drug use (yes/no), customised centile(6 groups), most recent going-to-sleep position (7 groups) and a stratification statement for study and site

*Adjusted OR is a multivariable model with each individual foetal movement variable added alone

**Multivariable model is a multivariable model including all foetal movement variables included in that column in the model. The individual frequency and strength variables are not included as they are accounted for in the combined variable, and uterine contractions were not included due to the lack of significance in the univariable analysis

Women who reported foetal hiccups in the last 2 weeks had decreased odds of late stillbirth (aOR = 0.45, 95% CI 0.36–0.58). Similarly, women who reported feeling multiple episodes of more vigorous than usual movements in the last 2 weeks were at decreased odds of having a late stillbirth (aOR = 0.67, 95% CI 0.52–0.87). However, those who reported more vigorous than usual movements on a single occasion in the last 2 weeks were at increased odds (aOR = 2.86, 95% CI 2.01–4.07) (Table 2).

### Patterns of foetal movements over gestation

**Fig. 1:**
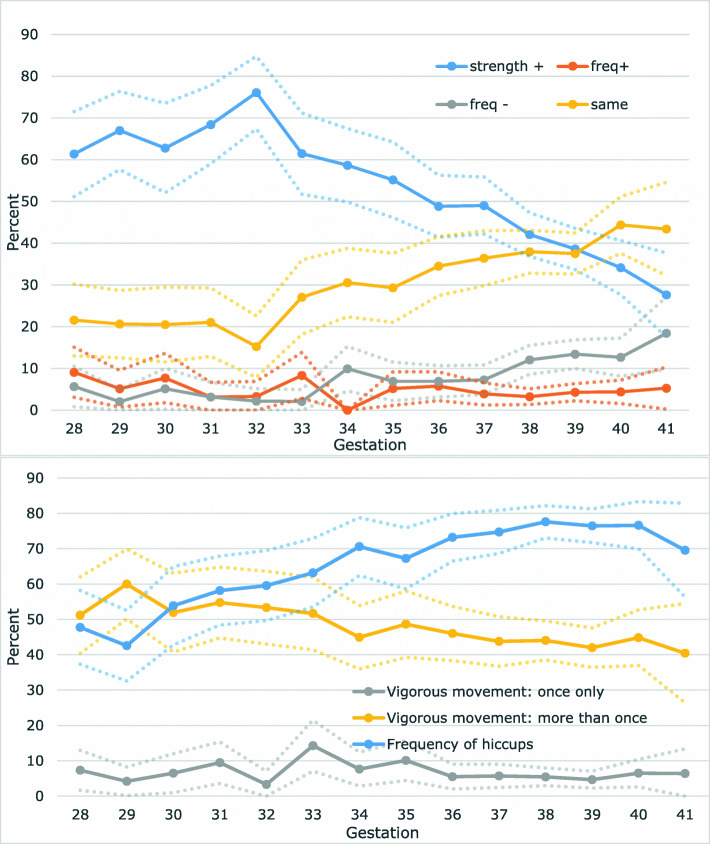
Prevalence of perception of foetal movements, hiccups and vigorous movements by gestational age in control women by gestational age. **A** Percentage of controls according to the prioritised strength and frequency of the foetal movements. **B** Percentage of controls with hiccups and vigorous foetal movements in the last 2 weeks. Dotted lines show 95% confidence intervals

The predominant pattern of foetal movements reported by controls in the preceding 2 weeks was an increase in strength (Fig. 1a). The prevalence of an increasing strength was relatively constant (> 60%) until approximately 33 weeks’ gestation after which the proportion reporting increased strength decreased but still remained the most frequent response until 39 weeks. Reporting of the perception of foetal hiccups increased as gestation advanced reaching a plateau at approximately 37 weeks’ gestation (Fig. 1b). Controls frequently reported multiple episodes of movements that were more vigorous than usual (over 50% during the very preterm period), although this decreased slightly as gestation increased. In contrast, an on-off occurrence of more vigorous than usual movements was reported by fewer than 10% of controls at almost all gestations. Corresponding data for cases is shown in Additional file 3: Fig S1.

### Interactions of foetal movements with gestation and obesity

In univariable analysis, we found a statistically significant interaction (*p* < 0.0001) between gestational age at interview and the prioritised strength-frequency foetal movement variable; this interaction remained statistically significant in the multivariable model (*p* = 0.01). The protective effect identified from the multivariable analysis of increased strength of movements decreased in magnitude as the pregnancy progressed aOR = 0.04 (95% CI 0.01–0.15) for very preterm, aOR = 0.13 (95% CI 0.07, 0.24) in preterm and aOR = 0.29 (95% CI 0.20–0.43) in term pregnancies (Table 3). The increased odds of late stillbirth associated with decreased frequency had a similar pattern, aOR = 6.98 (95% CI 1.63–29.84) in very preterm, aOR = 3.48 (95% CI 1.67–7.23) for preterm pregnancies and aOR = 1.95 (95% CI 1.33–2.86) at term. There was also a multivariable interaction (*p* = 0.05) between gestational age and more vigorous than usual movements. A single episode of more vigorous than usual movements had a higher odds of stillbirth at term gestation aOR = 3.78 (95% CI 2.35–6.08) compared to preterm aOR = 2.56 (95% CI 1.13–5.83) and very preterm aOR = 1.78 (95% CI 0.50–6.31) (Table 3). No interaction was detected between the gestational age group and the presence of hiccups (*p* = 0.62). Furthermore, no significant interactions were seen in terms of obesity vs. non-obesity and the prioritised strength-frequency foetal movement variable (*p* = 0.59), episodes of more vigorous movements than usual (*p* = 0.92) or hiccups (*p* = 0.17) (Table 4).

**Table 3:** Multivariable* odds ratios showing the association between foetal movement variables and late stillbirth by gestational age group

	Very preterm (≤ 32 weeks)	Preterm (33–36 weeks)	Term (37+ weeks)
	Cases, ***N*** = 114; controls, ***N*** = 323	Cases, ***N*** = 192; controls, ***N*** = 509	Cases, ***N*** = 401; controls, ***N*** = 897
**Combined strength and frequency of foetal movements in the last 2 weeks**			
Increased strength	**0.04 (0.01, 0.15)**	**0.13 (0.07, 0.24)**	**0.29 (0.20, 0.43)**
Increased frequency but not strength	**0.06 (0.01, 0.62)**	0.79 (0.27, 2.27)	0.58 (0.26, 1.26)
Decreased frequency	**6.98 (1.63, 29.84)**	**3.48 (1.67, 7.23)**	**1.95 (1.33, 2.86)**
Unsure of strength or frequency	0.25 (0.04, 1.49)	1.38 (0.44, 4.37)	0.95 (0.50, 1.82)
The same strength or frequency	1.00	1.00	1.00
**Vigorous Foetal movements in the last 2 weeks**			
Once	1.78 (0.50, 6.31)	**2.56 (1.13, 5.83)**	**3.78 (2.35, 6.08)**
More than once	0.66 (0.23, 1.88)	0.63 (0.36, 1.11)	0.66 (0.46, 0.93)
Never	1.00	1.00	1.00
**Hiccups in the last 2 weeks**			
Yes	0.61 (0.24, 1.58)	**0.33 (0.19, 0.56)**	**0.39 (0.29, 0.55)**
No	1.00	1.00	1.00
Unsure	0.83 (0.17, 3.91)	0.60 (0.21, 1.72)	0.98 (0.48, 1.98)

*Analyses adjusted for all confounding variables used in the base model in table 2 except gestation (continuous)

**Table 4:** Multivariable* odds ratios showing the association between foetal movement variables and late stillbirth by maternal obesity

	Obese (> 30 kg/m^**2**^)	Non-obese (≤ 30 kg/m^**2**^)
	Cases, ***N*** = 114; controls, ***N*** = 323	Cases, ***N*** = 192; controls, ***N*** = 509
**Combined strength and frequency of foetal movements in the last 2 weeks**		
Increased strength	**0.19 (0.10, 0.33)**	**0.20 (0.14, 0.29)**
Increased frequency but not strength	0.58 (0.23, 1.47)	**0.38 (0.17, 0.82)**
Decreased frequency	**3.01 (1.51, 5.99)**	**2.30 (1.62, 3.27)**
Unsure of strength or frequency	1.20 (0.47, 3.05)	0.81 (0.45, 1.46)
The same strength or frequency	1.00	1.00
**Vigorous Foetal movements in the last 2 weeks**		
Once	2.27 (0.98, 5.25)	**3.18 (2.11, 4.80)**
More than once	**0.50 (0.29, 0.84)**	**0.69 (0.50, 0.95)**
Never	1.00	1.00
**Hiccups in the last 2 weeks**		
Yes	**0.58 (0.36, 0.95)**	**0.40 (0.30, 0.54)**
No	1.00	1.00
Unsure	0.17 (0.05, 0.60)	1.16 (0.68, 1.98)

*Analyses adjusted for all confounding variables used in the base model in table 2

### Sensitivity analyses

Comparison of the models where each of the foetal movement variables was added individually to the potential confounders and the full multivariable model showed very little difference in odds ratios, suggesting that the effect was consistent without the Sydney study. Similarly, when the STARS study was excluded from the model, to assess the impact of missing data on foetal movement variables from that study, little change was seen in odds ratios (Additional file 1: Table S3). Additionally, removal of the strata statement from the model had little effect on the estimated parameters in the models.

The distribution of the cause of death (as determined by the PSANZ classification system [23]) differed in women who reported RFM compared to those who did not (chi-square = 24.60, *p* = 0.003) (Table 5). In cases with RFM, a greater proportion of late stillbirths were attributed to foetal growth restriction (15.9% in RFM vs. 10.3% with no RFM) and unexplained antepartum stillbirths (48.4% in RFM vs. 37.8% with no RFM) and a lower proportion of deaths due to antepartum haemorrhage (7.8% in RFM vs. 12.9% with no RFM) and hypoxic peripartum deaths (3.8% in RFM vs. 9.4% with no RFM). There were no statistical differences in the cause of late stillbirth in relation to vigorous movements (Additional file 1: Table S4).

**Table 5:** Association of cause of death (PSANZ Classification) in stillbirths by maternal perception of reduced foetal movements

PSANZ code for cause of death	Reduced foetal movements			
	Chi-square = 24.60, ***p*** = 0.003			
	Yes	%	No	%
1. Congenital abnormality	1	0.3%	1	0.4%
2. Perinatal infection	17	4.6%	13	5.6%
3. Hypertension	16	4.3%	11	4.7%
4. Antepartum haemorrhage (APH)	29	7.8%	30	12.9%
5. Maternal conditions	20	5.4%	17	7.3%
6. Specific perinatal conditions	36	9.7%	24	10.3%
7. Hypoxic peripartum death	14	3.8%	22	9.4%
8. Foetal growth restriction (FGR)	59	15.9%	24	10.3%
9. Spontaneous preterm (< 37 weeks gestation)	0	0.0%	3	1.3%
10. Unexplained antepartum death	180	48.4%	88	37.8%

## Discussion

Our findings confirm that women can expect to feel increasingly stronger movements through the third trimester of pregnancy, and can be reassured by perception of regular periods of vigorous movements and foetal hiccups. Conversely, perception of decreased frequency of foetal movements in late pregnancy is associated with increased odds of late stillbirth at all gestations but more so early in the third trimester. Our findings also suggest that at term (37 weeks’ gestation or later), a single isolated occurrence of more vigorous movements is associated with late stillbirth.

A strength of this study is that it had a large sample size and extensive collection of pregnancy-related variables, containing data from across several countries. This has allowed exploration of the interactions between gestation, obesity and changes in foetal activity which individual studies were not powered to do, providing valuable additional information. Analysis was also able to be carried out to investigate the classification of cause of death according to RFM and episodes of increased foetal movements.

The study has by its nature some limitations; case-control studies are subject to the potential of recall bias. The risk of this was reduced in these studies by the use of interviewer-administered questionnaires in four of the studies included, which contained no hypothesis about the potential association of various patterns of movements. The potential for selection bias also exists; however, the reasons for this would likely vary across countries, yet the prevalence of foetal movement variables was relatively consistent between studies. The risk of bias assessment using the ROBINS-E tool has been reported previously, and showed that 4 of the 5 studies had a moderate risk of bias, i.e. does not provide the level of evidence of a randomised trial, and the internet study a serious risk of bias. The sensitivity analyses however did not show any significant changes in the results when various studies were excluded from the analyses, giving us confidence in the findings.

Historically, stillbirth research has focused on a decrease in the frequency of movements, with studies and campaigns based around kick counts [9, 24]. The data presented here show that women should expect that the strength of foetal movement should remain at least as strong throughout late pregnancy but should, more often than not, increase in strength until approximately 37 weeks’ gestation when it plateaus. Women perceive these changes in strength differently, and some (40–50% as gestation progresses) may not feel stronger movement. The perception of increasing strength of movement may simply be due to increasing foetal size, and relatively limited space making movement more perceptible. Nevertheless, this is regarded as part of normal foetal development. Given that increased strength of movements is the most commonly reported pattern, feeling decreased movement becomes an even more important consideration. The risk associated with RFM has previously been calculated in comparison with women who detect no change [5–7], rather than those who feel a gradual increase in foetal movements.

Notably, this IPD has also shown that gestational age at the time of assessment of foetal movements is important in assessing the significance of changes in foetal movement patterns. Increasing strength of movements is most important before 37 weeks’ gestation as the magnitude of the reduced odds of late stillbirth in the presence of increased strength of foetal movements is greater than after 37 weeks. This has clinical implications for the assessment of women attending with RFM before 37 weeks, as the association with stillbirth during this period of gestation is stronger than at term. The association between RFM and FGR suggests particular attention should be made to excluding FGR and placental dysfunction in those pregnancies before 37 weeks’ gestation who present with RFM.

Our findings suggest that maternal perception of foetal hiccups is common and associated with reduced odds of late stillbirth. The phenomenon of foetal hiccups was first reported by Ferroni in 1899 [25] and considered to be part of normal foetal development [26]. The prevalence of hiccups in this analysis increased through to approximately 37 weeks’ gestation, which is divergent to other reports that suggest foetal hiccups are more prevalent earlier in pregnancy and decrease as pregnancy progresses [27, 28]. As with general foetal movements, increased maternal perception of foetal hiccups near term may be due to greater foetal size, changes in foetal breathing or neurodevelopment or may reflect increased maternal recognition of the sensation. The data from this IPD indicates that foetal hiccups are a normal part of pregnancy and are not associated with increased odds of stillbirth.

The AFFIRM and Mindfetalness trials have raised debate around the clinical usefulness of foetal movement awareness to prevent stillbirth. Critically, the AFFIRM study protocol which reported no reduction in the rate of late stillbirth (aOR 0.90, 95% confidence interval (CI) 0.75–1.07) [4] recommended intervention for RFM after 37 weeks’ gestation, particularly when recurrent at term. Our analysis demonstrates that this recommendation coincided with the lowest, though still increased, odds of stillbirth associated with RFM. The Mindfetalness trial employed a structured approach to awareness of foetal activity [8] and found no difference in the primary outcome of the number of babies born with an Apgar score < 7 [8]. Importantly, both AFFIRM and Mindfetalness identified that the investigation and subsequent management of RFM reduces the proportion of SGA infants at birth compared to the control groups. This is consistent with the association between RFM and late stillbirths due to foetal growth restriction. McCarthy et al. reported that women presenting with RFM have a higher burden of care, including increased rates of induction, admission to neonatal units and higher levels of surveillance [29]. Our findings suggest that women with RFM should be assessed to exclude foetal compromise and FGR rather than receiving intervention for RFM alone to focus intervention on those most likely to benefit.

Importantly, our analysis found no interaction between the combined strength and frequency variable and maternal obesity. A systematic review of obesity and foetal movements identified limited data and reported that maternal body size was not associated with altered ability to perceive foetal movements (4 studies of 95 women; very low-quality evidence) [14]. In a further study, maternal reporting of foetal movement strength and frequency was not different in relation to obesity, highlighting that maternal BMI is not a barrier to the detection of foetal movements and the clinical importance of a presentation with foetal movement concerns is not diminished by maternal body size [15]. As such, all women regardless of BMI, attending with concerns about foetal movements, should be treated the same. The increased risk of stillbirth in relation to obesity is likely to be multifactorial; previous studies have continued to show an increased risk associated with obesity even after controlling for factors such as diabetes and pre-eclampsia [19, 30].

The most difficult finding to interpret from this IPD meta-analysis has been that of foetal movements that are more vigorous than usual. Nearly half of women report this occurring on more than one occasion which appears to be protective of late stillbirth. Only women who report an isolated instance of more vigorous than usual foetal movements have increased odds of late stillbirth. The prevalence of a single event of vigorous movements in our data (18% in cases vs. 6% in controls) corresponds to the data from other studies which suggest an incidence of 10% amongst late stillbirths [11]. It has been suggested that such excessively vigorous movements could be related to foetal seizure activity or umbilical cord compression or entanglement [31]. Women who have a late stillbirth and report one instance of vigorous foetal movements often describe the movements as ‘crazy’ and ‘wild’ [10]. The distinction between ongoing vigorous movements and a single episode of exaggerated foetal activity is difficult and can only be achieved in retrospect, as exemplified by two cohort studies of women presenting with increased foetal movements which both found no association with adverse outcome [32, 33]. However, our analysis suggests that the pattern of vigorous foetal movements needs to be considered in light of the gestation, with the highest odds of stillbirth in women with a single episode of increased foetal movements, after 37 weeks’ gestation. Therefore, further research would be valuable in relation to increased movements. The ideal design would come from women who had previously experienced vigorous movements both as part of a pregnancy with a live born and as part of another pregnancy resulting in a stillbirth, such a study would be difficult to undertake in relation to sample size and recall bias. A follow-up study to STARS—the Pregnancy Research Project—is currently collecting such data (O’Brien, personal communication).

## Conclusion

In conclusion, this analysis has shown that increasing strength of movements throughout the third trimester is the most typical pattern of foetal movements, alongside the experience of foetal hiccups; this should be considered part of normal foetal development and is associated with decreased odds of late stillbirth. Maternal obesity should not be interpreted as a reason for maternal perception of reduced movements. A decrease in the frequency of foetal movements in the third trimester is of concern, particularly at earlier gestations and thus merits investigation to exclude acute and/or chronic foetal compromise. Experiencing a single episode of unusually vigorous movements particularly at term is also associated with stillbirth, but remains an area requiring further research before it can be decided whether any clinical recommendations can be made.

## Supplementary Information

**Additional file 1: Table S1.** Description of studies included in the CRIBBS Individual Pooled Meta-Analysis. **Table S2.** Breakdown of numbers in the IPD analysis by individual study for fetal movement variables. **Table S3.** Sensitivity analysis excluding STARS study from the multivariable model.

**Additional file 2.** SAS code for multivariable model to assess the independent effects of perception of fetal movements, with explanation of purpose of lines of code.

**Additional file 3: Figure S1.** Prevalence of change perception of fetal movements, hiccups and vigorous movements by gestational age in women who experienced a stillbirth. A) Percentage of cases according to the prioritised strength and frequency of fetal movements, B) Percentage of cases with hiccups and vigorous fetal movements in the last 2 weeks.

## Data Availability

Data cannot be shared publicly beyond the Collaborative Individual Participant Data Meta-analysis of Sleep and Stillbirth (CRIBSS) group as no individual participating study obtained consent from participants to make the data publically available. Furthermore, because stillbirth is uncommon, there is potential for participants to be identifiable. Contact for the CRIBSS Data Access Committee can be made through the corresponding author.

## Abbreviations

aOR: Adjusted odds ratio
BMI: Body mass index
cRCT: Cluster randomised control trial
CRIBSS: Collaborative Individual Participant Data Meta-analysis of Sleep and Stillbirth
IPD: Individual participant data meta-analysis
MCSS: Multi-Centre Stillbirth Study
MINESS: Midlands and North East Stillbirth Study
OR: Odds ratio
RFM: Reduced foetal movements
SSS: Sydney Stillbirth Study
STARS: *Study* of Trends and Associated Risks for *Stillbirth*
TASS: The Auckland Stillbirth Study
