# Supplementary material for: A better understanding of the association between maternal perception of foetal movements and late stillbirth—findings from an individual participant data meta-analysis
Source: BMC Med. 2021 Nov 15;19:267. doi: 10.1186/s12916-021-02140-z (PMC8591897; doi:10.1186/s12916-021-02140-z)
Supplement: Supplementary file 1 — Additional file 1: Table S1. Description of studies included in the CRIBBS Individual Pooled Meta-Analysis. Table S2. Breakdown of numbers in the IPD analysis by individual study for fetal movement variables. Table S3. Sensitivity analysis excluding STARS study from the multivariable model. [file 12916_2021_2140_MOESM1_ESM.docx]

**Additional File 1**

**Supplementary Table 1. Description of studies included in the CRIBBS Individual Pooled Meta-Analysis**

| **Study Location** | **Recruitment Period** | **Study Design** | **Population** | **Number of Cases and Controls in Original Study Population** | **Data Collection** | **Risk of Bias** |
| --- | --- | --- | --- | --- | --- | --- |
| Auckland, New Zealand | July 2006 to June 2009 | Prospective population-based case–control study | Non-anomalous singleton pregnancy, ≥28 weeks' gestation, from three health regions in Auckland, New Zealand | 155 cases,  310 controls | Interview and clinical records | Moderate |
| Sydney, Australia | January 2006 to December 2011 | Prospective population-based case–control study | Non-anomalous singleton pregnancy, ≥32 weeks' gestation, from nine tertiary maternity facilities in metropolitan Sydney, Australia | 103 cases,  192 controls | Interview and clinical records | Moderate |
| New Zealand | February 2012 to December 2015 | Prospective population-based case–control study | Non-anomalous singleton pregnancy, ≥28 weeks' gestation, from seven health regions throughout New Zealand | 164 cases,  569 controls | Interview and clinical records | Moderate |
| United Kingdom | April 2014 to March 2016 | Prospective population-based case–control study | Non-anomalous singleton pregnancy, ≥28 weeks' gestation, from 41 maternity facilities in the United Kingdom | 291 cases,  733 controls | Interview and clinical records | Moderate |
| International  USA n=447 (71%)  Canada n=44 (7%)  UK n=95 (15%)  Other n=40 (6%) | September 2012 to August 2014 | Nested case–control study with an uncontrolled cohort | Singleton pregnancy, ≥28 weeks' gestation, fluent in English, from 16 high, middle, and low income countries | 153 cases,  480 controls | Online survey | Serious |

**Supplementary Table 2. Breakdown of numbers in the IPD analysis by individual study for fetal movement variables**

|  | **TASS** |  |  | **SYDNEY** |  |  | **MCSS** |  |  | **MINESS** |  |  | **STARS** |  |  |
| --- | --- | --- | --- | --- | --- | --- | --- | --- | --- | --- | --- | --- | --- | --- | --- |
|  |  |  |  |  |  |  |  |  |  |  |  |  |  |  |  |
|  | **Case** | **Control** | **OR (95% CI)** | **Case** | **Control** | **OR (95% CI)** | **Case** | **Control** | **OR (95% CI)** | **Case** | **Control** | **OR (95% CI)** | **Case** | **Control** | **OR (95% CI)** |
|  | **n** | **n** |  | **n** | **n** |  | **n** | **n** |  | **n** | **n** |  | **n** | **n** |  |
| **Total number of participants** | 155 | 310 |  | 103 | 192 |  | 164 | 569 |  | 291 | 733 |  | 153 | 480 |  |
| **Total number of participants in IPD dataset (i.e. after exclusions)** | 155 | 304 |  | 103 | 192 |  | 163 | 560 |  | 288 | 733 |  | 142 | 468 |  |
|  |  |  |  |  |  |  |  |  |  |  |  |  |  |  |  |
| **Strength of movements in last 2 weeks: 4 categories** |  |  |  |  |  |  |  |  |  |  |  |  |  |  |  |
| Increased | 16 | 124 | 0.19 (0.11, 0.35) | 8 | 31 | 0.45 (0.20, 1.03) | 32 | 330 | 0.22 (0.14, 0.34) | 53 | 455 | 0.15 (0.11, 0.22) | 18 | 130 | 0.38 (0.21, 0.67) |
| Decreased | 30 | 21 | 2.11 (1.22, 3.95) | 5 | 8 | 1.09 (0.35, 3.46) | 47 | 45 | 2.35 (1.45, 3.82) | 62 | 50 | 1.61 (1.05, 2.46) | 58 | 56 | 2.83 (1.78, 4.49) |
| No change | 76 | 112 | 1 | 75 | 131 | 1 | 80 | 180 | 1 | 153 | 198 | 1 | 66 | 180 | 1 |
| Unsure | 33 | 53 | 0.92 (0.54, 1.55) | 15 | 22 | 1.19 (0.58, 2.43) | 5 | 13 | 0.87 (0.30, 2.51) | 22 | 22 | 1.29 (0.69, 2.42) | 4 | 11 | 0.99 (0.31, 3.22) |
|  |  |  |  |  |  |  |  |  |  |  |  |  |  |  |  |
| **Frequency of movements in last 2 weeks: 4 categories** |  |  |  |  |  |  |  |  |  |  |  |  |  |  |  |
| Increased | 13 | 88 | 0.26 (0.14, 0.50) | 6 | 17 | 0.63 (0.24, 1.65) | 21 | 221 | 0.31 (0.18, 0.51) | 37 | 254 | 0.38 (0.26, 0.56) | 7 | 75 | 0.32 (0.14, 0.73) |
| Decreased | 45 | 36 | 2.22 (1.32, 3.74) | 9 | 13 | 1.23 (0.50, 3.00) | 62 | 83 | 2.41 (1.59, 3.65) | 86 | 63 | 3.57 (2.45, 5.19) | 73 | 76 | 3.30 (2.16, 5.03) |
| No change | 76 | 135 | 1 | 74 | 131 | 1 | 79 | 255 | 1 | 152 | 397 | 1 | 65 | 223 | 1 |
| Unsure | 21 | 51 | 0.73 (0.41, 1.31) | 14 | 31 | 0.80 (0.40, 1.60) | 2 | 10 | 0.65 (0.14, 3.01) | 15 | 17 | 2.31 (1.12, 4.73) | 1 | 6 | 0.57 (0.07, 4.84) |
|  |  |  |  |  |  |  |  |  |  |  |  |  |  |  |  |
| **How often was baby more vigorous than usual in last 2 weeks: 3 categories** |  |  |  |  |  |  |  |  |  |  |  |  |  |  |  |
| Once | 32 | 16 | 4.17 (2.18, 7.96) | N/A | N/A | N/A | 22 | 34 | 1.59 (0.89, 2.86) | 41 | 50 | 1.47 (0.94, 2.31) | 42 | 24 | 4.24 (2.36, 7.62) |
| More than once | 25 | 91 | 0.57 (0.35, 0.95) |  |  |  | 40 | 280 | 0.35 (0.23, 0.53) | 68 | 354 | 0.34 (0.25, 0.47) | 37 | 189 | 0.47 (0.30, 0.76) |
| Never | 97 | 202 | 1 |  |  |  | 97 | 239 | 1 | 182 | 326 | 1 | 59 | 143 | 1 |
|  |  |  |  |  |  |  |  |  |  |  |  |  |  |  |  |
| **During the last 2 weeks/ this pregnancy did you feel your baby having hiccups?** |  |  |  |  |  |  |  |  |  |  |  |  |  |  |  |
| Yes | 51 | 199 | 0.26 (0.17, 0.40) | N/A | N/A | N/A | 77 | 406 | 0.39 (0.27, 0.57) | 127 | 460 | 0.41 (0.31, 0.55) | 107 | 305 | 0.85 (0.52, 1.39) |
| No | 98 | 100 | 1 |  |  |  | 68 | 140 | 1 | 141 | 209 | 1 | 28 | 68 | 1 |
| Unsure | 6 | 10 | 0.61 (0.21, 1.75) |  |  |  | 17 | 22 | 1.59 (0.79, 3.19) | 23 | 62 | 0.55 (0.33, 0.93) | 6 | 8 | 1.82 (0.58, 5.73) |
|  |  |  |  |  |  |  |  |  |  |  |  |  |  |  |  |
| **During the last two weeks, did you feel uterine contractions (tightenings/pre-labour contractions/ Braxton Hicks contractions/ false labour)?** |  |  |  |  |  |  |  |  |  |  |  |  |  |  |  |
| Yes | 94 | 189 | 0.99 (0.67, 1.46) | N/A | N/A | N/A | 67 | 227 | 1.05 (0.74, 1.49) | 94 | 241 | 0.97 (0.73, 1.30) | N/A | N/A | N/A |
| No | 61 | 121 | 1 |  |  |  | 96 | 341 | 1 | 197 | 490 | 1 |  |  |  |
|  |  |  |  |  |  |  |  |  |  |  |  |  |  |  |  |
| **Combination of strength and frequency changes in the last 2 weeks (prioritised variable)** | | | | | | | | | | | | | | | |
| Missing | 0 | 0 |  | 0 | 0 |  | 0 | 0 |  | 0 | 2 |  | 7 | 99 |  |
| Increased strength | 16 | 124 | 0.18 (0.10, 0.33) | 8 | 31 | 0.45 (0.20, 1.04) | 32 | 330 | 0.21 (0.13, 0.33) | 53 | 455 | 0.18 (0.13, 0.26) | 18 | 130 | 0.39 (0.22, 0.69) |
| Increased frequency but not strength | 6 | 30 | 0.28 (0.11, 0.70) | 1 | 4 | 0.44 (0.05, 4.00) | 9 | 29 | 0.67 (0.30, 1.50) | 8 | 22 | 0.57 (0.25, 1.31) | 1 | 14 | 0.20 (0.03, 1.55) |
| Decreased frequency | 45 | 29 | 2.15 (1.22, 3.76) | 9 | 9 | 1.76 (0.67, 4.62) | 56 | 58 | 2.08 (1.30, 3.34) | 79 | 36 | 3.42 (2.18, 5.37) | 69 | 67 | 2.87 (1.83, 4.52) |
| Unsure of strength or frequency | 20 | 33 | 0.84 (0.44, 1.58) | 11 | 18 | 1.07 (0.48, 2.40) | 3 | 14 | 0.46 (0.13, 1.67) | 22 | 17 | 2.02 (1.03, 3.94) | 1 | 11 | 0.25 (0.03, 2.01) |
| Same strength or frequency | 68 | 94 | 1 | 74 | 130 | 1 | 64 | 138 | 1 | 129 | 201 | 1 | 57 | 159 | 1 |

**Supplementary Table 3. Sensitivity analysis excluding STARS study from the multivariable model**

|  | **Multivariable OR**  **(95% CI) (N = 2149)** |
| --- | --- |
|  |  |
| **Combination of strength and frequency changes in the last 2 weeks (prioritised variable)** |  |
| Increased strength | **0.17 (0.13, 0.24)** |
| Increased frequency but not strength | **0.51 (0.28, 0.91)** |
| Decreased frequency | **2.40 (1.71, 3.37)** |
| Unsure of strength or frequency | 0.84 (0.51, 1.38) |
| Same strength or frequency | 1 |
|  |  |
| **How often was baby more vigorous than usual in last 2 weeks: 3 categories** |  |
| Once | **2.73 (1.84, 4.07)** |
| More than once | **0.68 (0.51, 0.91)** |
| Never | 1 |
|  |  |
| **During the last 2 weeks/ this pregnancy did you feel your baby having hiccups?** |  |
| Yes | **0.43 (0.33, 0.56)** |
| No | 1 |
| Unsure | 0.82 (0.50, 1.35) |

**Supplementary Table 4. Association of Cause of Death (PSANZ Classification) in stillbirths by maternal perception of vigorous fetal movements.**

| **PSANZ code for cause of death:** | **Once** | **%** | **More than once** | **%** | **Never** | **%** |
| --- | --- | --- | --- | --- | --- | --- |
| 1 Congenital abnormality | 0 | 0.0% | 0 | 0.0% | 2 | 0.5% |
| 2 Perinatal infection | 4 | 4.3% | 7 | 5.3% | 19 | 5.1% |
| 3 Hypertension | 7 | 7.4% | 8 | 6.1% | 12 | 3.2% |
| 4 Antepartum haemorrhage (APH) | 6 | 6.4% | 17 | 12.9% | 35 | 9.4% |
| 5 Maternal conditions | 5 | 5.3% | 11 | 8.3% | 21 | 5.6% |
| 6 Specific perinatal conditions | 14 | 14.9% | 12 | 9.1% | 34 | 9.1% |
| 7 Hypoxic peripartum death | 4 | 4.3% | 6 | 4.5% | 26 | 7.0% |
| 8 Fetal Growth Restriction (FGR) | 11 | 11.7% | 14 | 10.6% | 58 | 15.5% |
| 9 Spontaneous preterm (<37 weeks gestation) | 0 | 0.0% | 1 | 0.8% | 2 | 0.5% |
| 10 Unexplained antepartum death | 43 | 45.7% | 56 | 42.4% | 164 | 44.0% |
