# Supplementary material for: A better understanding of the association between maternal perception of foetal movements and late stillbirth—findings from an individual participant data meta-analysis
Source: BMC Med. 2021 Nov 15;19:267. doi: 10.1186/s12916-021-02140-z (PMC8591897; doi:10.1186/s12916-021-02140-z)
Supplement: Supplementary file 2 — Additional file 2. SAS code for multivariable model to assess the independent effects of perception of fetal movements, with explanation of purpose of lines of code. [file 12916_2021_2140_MOESM2_ESM.docx]

**Additional File 2: SAS code for multivariable model to assess the independent effects of perception of fetal movements, with explanation of purpose of lines of code.**

**proc** **logistic** data= Cribss3; Calls the logistic regression procedure and defines the dataset to be used

class This class statement identifies categorical variables for the model statement

C1_age_group (ref='4.30-34') Categorical variables are: Maternal age

C13_mat_ethni_group (ref= '1.White') Ethnicity

C23parity_group (ref='2.1-2') Parity

C15_mat_edu_99ismissing (ref='3') Maternal education

C19_marital_status_2g (ref='1') Marital status

C22_mat_htn_DM (ref='0') Maternal Hypertension or Diabetes

C31_smking_status_2g (ref='2') Maternal smoking

C39_recreation_drugs (ref='0') Recreational drugs

C163cus_centil_6g (ref='5.75-89.99') Birthweight percentile

C84_IPD_gosleep_mst_recen (ref='1') Maternal sleep position

FM_strength_freq_pri (ref='5.Same') Prioritised fetal movement variable

C127_vigours_FM_freq_lat2wk (ref='3') Vigorous movements

C128_hiccups_last2wk_yn (ref='No') Fetal hiccups

/param=ref; Statement to provide parameter estimates compared to reference group

Model C3_case_con (event='1.Case')= Model statement to fit model, categorical variables are as defined in above class statement

C1_age_group

c2mat_frst_BMI BMI included as a continuous variable

C13_mat_ethni_group

C23parity_group

C15_mat_edu_99ismissing

C19_marital_status_2g

C22_mat_htn_DM

C31_smking_status_2g

C39_recreation_drugs

C163cus_centil_6g

C84_IPD_gosleep_mst_recen

C9_matching_ges_wk Gestational age as a continuous variable

FM_strength_freq_pri

C127_vigours_FM_freq_lat2wk

C128_hiccups_last2wk_yn /maxiter = **100**;

strata C2_orig_study C4_study_site; Strata statement to allow for both the individual study effect and sites within studies

where C2_orig_study ne "2.Sydney"; Excludes the Sydney Study from this model due to missing data

**run**;
