# Supplementary material for: A better understanding of the association between maternal perception of foetal movements and late stillbirth—findings from an individual participant data meta-analysis
Source: BMC Med. 2021 Nov 15;19:267. doi: 10.1186/s12916-021-02140-z (PMC8591897; doi:10.1186/s12916-021-02140-z)
Supplement: Supplementary file 3 — Additional file 3: Figure S1. Prevalence of change perception of fetal movements, hiccups and vigorous movements by gestational age in women who experienced a stillbirth. A) Percentage of cases according to the prioritised strength and frequency of fetal movements, B) Percentage of cases with hiccups and vigorous fetal movements in the last 2 weeks. [file 12916_2021_2140_MOESM3_ESM.docx]

**Additional File 3: Figure S1**. Prevalence of change perception of fetal movements, hiccups and vigorous movements by gestational age in women who experienced a stillbirth. A) Percentage of cases according to the prioritised strength and frequency of fetal movements, B) Percentage of cases with hiccups and vigorous fetal movements in the last 2 weeks. Dotted lines show 95% confidence intervals.
